# Dr. S. P. Hullihen

**Published:** 1857-04

**Authors:** 


OBITUARY.—Dr. S. P. HULLIHEN died on Friday, 27th March, at his residence, in the city of Wheeling, of pneumonia, after a painful illness of some eight or ten days. Few men in the walks of professional life, have more fully illustrated the rare combination of high moral and social virtues, with eminent professional acquirements and most laborious devotion to science.

In no community has the death of a professional man produced a profounder grief, every citizen felt that a calamity had fallen upon him, and upon his city. The poor and suffering especially felt that more than a friend had gone from them; his noble heart always beat responsive to the claims of humanity, among all states and conditions of society. His sympathies were ever excited by the poverty and sufferings of his fellow man, and his hand extended to relieve the one, and his skill exerted for the mitigation of the other. As a practical surgeon, Dr. H. had few superiors, and many of his operations exhibited the genius and skill of professional mind of the highest order; in the various branches of dental science, he was justly distinguished.

No higher evidence could be afforded of the professional and social estimation in which Dr. H. was held by his fellow citizens, than the spontaneous expression of their feelings upon the announcement of his death. The medical faculty of Ohio county, the council of the city, the trustees of the Wheeling hospital, of which he was one of the founders, and a large meeting of the citizens assembled at the court house, severally, expressed, in appropriate resolutions, a deep sense of the personal and professional worth of the deceased, and their profound grief under the bereavement they had sustained in his death. At the meeting of the citizens it was determined to erect a magnificent monument to his memory.

On the day of the funeral until the afternoon hour appointed for the removal of the body to its last resting place, it lay in state in one of his parlors, and was visited by hundreds, anxious to take a farewell look at a benefactor and friend, and as the funeral *cortegé* passed on through the streets to Mt. Woods' Cemetery, it seemed that the whole city had fallen into line in the procession, which extended for more than a mile.

Thus do "we all fade as a leaf," illustrating every day, "what shadows we are and what shadows we pursue."—"No man can give a ransom for his brother, or redeem his soul from death."

Dr. H. has been intimately associated with the efforts made to advance Dental Science ; he was ever a warm friend of the Baltimore College of Dental Surgery, and an active member of the American Society of Surgeon Dentists. We hope in a future number to be able to furnish an extended memoir of this distiguished gentleman. His death comes upon us suddenly, and while we were making up the last pages of the journal.—*Requiescat in pace.*

